# Septo–hippocampal interaction

**DOI:** 10.1007/s00441-017-2745-2

**Published:** 2017-12-18

**Authors:** Christina Müller, Stefan Remy

**Affiliations:** 1 Neuronal Networks Group, German Center for Neurodegenerative Diseases in the Helmholtz Association (DZNE e.V.), Bonn, Germany; 2 Department of Epileptology, University of Bonn, Bonn, Germany

**Keywords:** Medial septum, Hippocampus, Theta oscillation, Behavior, Locomotion

## Abstract

The septo–hippocampal pathway adjusts CA1 network excitability to different behavioral states and is crucially involved in theta rhythmogenesis. In the medial septum, cholinergic, glutamatergic and GABAergic neurons form a highly interconnected local network. Neurons of these three classes project to glutamatergic pyramidal neurons and different subsets of GABAergic neurons in the hippocampal CA1 region. From there, GABAergic neurons project back to the medial septum and form a feedback loop between the two remote brain areas. In vivo, the firing of GABAergic medial septal neurons is theta modulated, while theta modulation is not observed in cholinergic neurons. One prominent feature of glutamatergic neurons is the correlation of their firing rates to the animals running speed. The cellular diversity, the high local interconnectivity and different activity patterns of medial septal neurons during different behaviors complicate the functional dissection of this network. New technical advances help to define specific functions of individual cell classes. In this review, we seek to highlight recent findings and elucidate functional implications of the septo-hippocampal connectivity on the microcircuit scale.

## Introduction

Knowing the structural and functional connectivity of specific brain regions is essential to understand the link between behavior and neuronal activity. A highly interconnected brain region contains the medial septum and the diagonal band of Broca (MSDB) within the basal forebrain. Among others, it receives inputs from the hippocampus, the amygdala, the supra-mammillary nuclei, the thalamus and the ventral tegmental area and projects to the entire hippocampal formation, the amygdala, the ventral tegmental area and the hypothalamus (Fuhrmann et al. 2015; Swanson and Cowan 1979). Thus, the MSDB can be regarded as a pivotal node within an ascending pathway from the brainstem and the hypothalamus that conveys sensory and motor information to the limbic system (Bland and Oddie 2001).

**Fig. 1:**
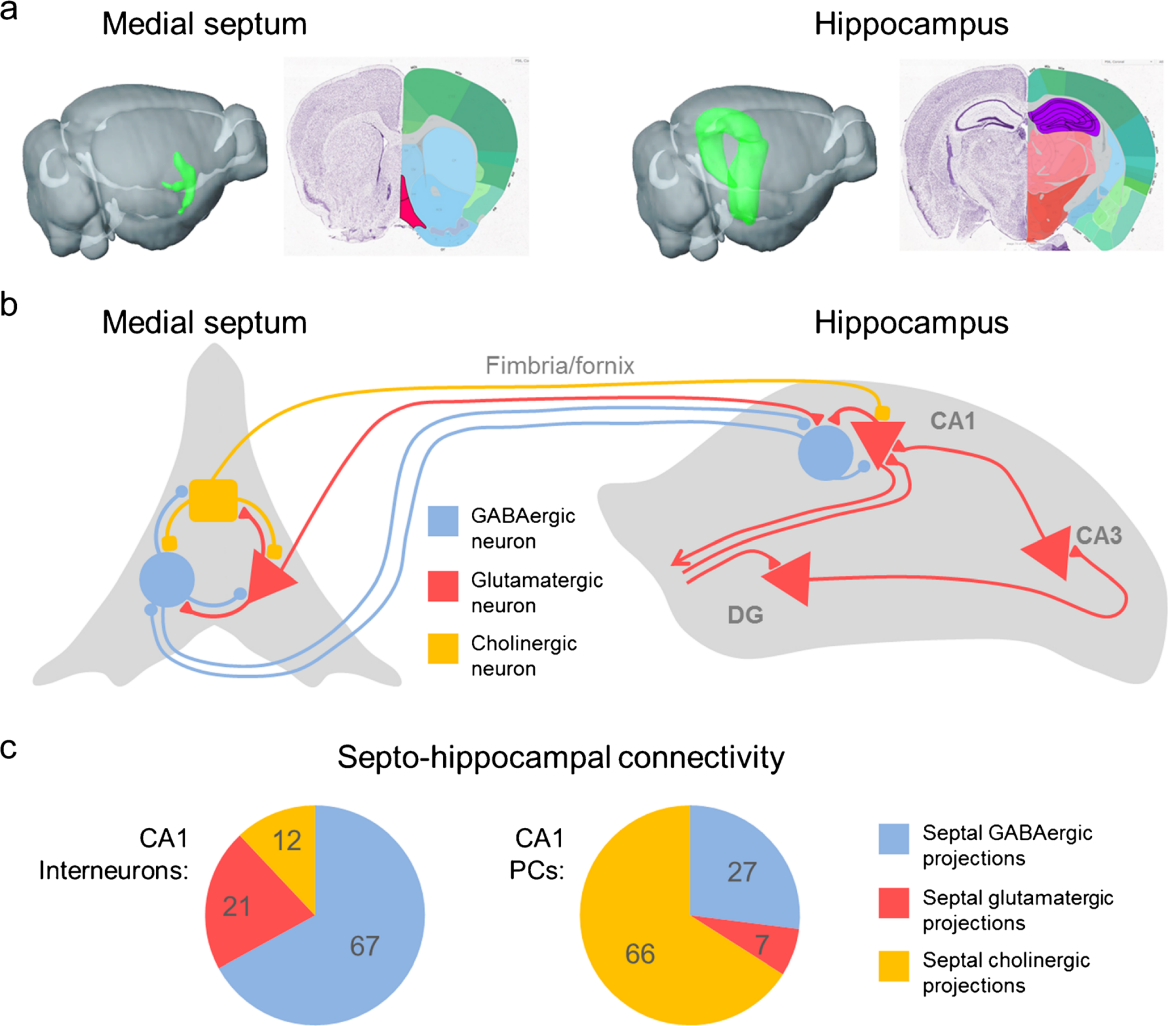
The septo-hippocampal connections.** a** From* left* to* right*: location of the medial septum (green structure) in the whole mouse brain and in a coronal section, bregma 1.045 mm (*red structure*). Location of the hippocampus (*green structure*) in the whole mouse brain and in a coronal section, bregma −1.955 mm (*violet structure*); image credit: Allen Institute. **b** Simplified schematic drawing of septo-hippocampal connectivity focusing on medial septal connections to CA1. GABAergic neurons are depicted in* blue*, glutamatergic in* red* and cholinergic in* yellow*. **c** Proportion of GABAergic, glutamatergic and cholinergic projections terminating on CA1 interneurons and CA1 pyramidal neurons (Sun et al. 2014)

Anatomically, the MSDB can be divided into the more dorsally located medial septal nucleus and the ventrally located diagonal band (Kiss et al. 1990a, b). In this region, GABAergic (immunopositive for GAD), cholinergic (immunopositive for ChAT) and glutamatergic neurons (immunopositive for VGluT1 and/or VGluT2; Frotscher and Léránth 1985; Hajszan et al. 2004; Kiss et al. 1990a, b) are found. Also, a subpopulation of neurons expressing both GAD and ChAT has been described (Sotty et al. 2003). The three major cell types in the medial septum are locally interconnected, giving rise to a dense local network (Leao et al. 2014). Activation of cholinergic neurons in the medial septum results in slow excitation of glutamatergic neurons. Glutamatergic neurons provide strong and comparably fast excitatory drive onto the other two cell types and form recurrent connections (Manseau et al. 2005), while local GABAergic connections synchronize the septal network to pace the rhythm of theta oscillations (Fuhrmann et al. 2015; Hangya et al. 2009; Huh et al. 2010). This strong local interconnectivity, however, makes it difficult to use pharmacological and cell type-specific manipulations within the medial septum to carve out the net effect of individual efferent projections on cellular activity in downstream regions. One major projection from septal GABAergic, glutamatergic and cholinergic neurons extends through the fimbria/fornix fiber bundle to the hippocampus. Septo–hippocampal GABAergic projections terminate predominantly on GABAergic neurons in the hippocampus (Freund and Antal 1988). Similarly, the main targets of septal–hippocampal glutamatergic projections are GABAergic neurons. In contrast, the main targets of septal cholinergic projections to the hippocampus are primarily pyramidal neurons (see Fig. 1; Sun et al. 2014).

The hippocampus, as part of the temporal lobe, is involved in episodic memory and plays an important role in spatial navigation (Anderson et al. 2007; Eichenbaum 2017; O’Keefe and Recce 1993; Rivas et al. 1996; Whishaw and Vanderwolf 1973). Multimodal sensory information, processed by the entorhinal cortex, enters the hippocampus via two pathways: the trisynaptic loop from the dentate gyrus, CA3 and CA1 and then back to the entorhinal cortex and the monosynaptic, or temporoammonic, pathway, from the entorhinal cortex directly to CA1 (Amaral and Witter 1989). It has been hypothesized that the hippocampal sub-regions, from CA1 to CA3 and the DG, are allocated with distinct functions during memory formation (Leutgeb and Leutgeb 2007). Furthermore, CA1 pyramidal neurons encode the animal’s location in space by increasing their firing probability, when the animal moves to a certain location in the environment. Thus, these spatially tuned CA1 pyramidal neurons are called place cells (O’Keefe 1979).

The hippocampus and the medial septum are connected reciprocally and neurons in both regions show correlated rhythmic activity in the theta frequency band (Dragoi et al. 1999; King et al. 1998). Theta oscillations are local field potential (LFP) fluctuations between 4 and 12 Hz, which reflect rhythmic changes of the synaptic inputs to the hippocampus (Bland and Oddie 2001, Buzsáki 2002). It was suggested that theta oscillations in the CA1 sub-region of the hippocampus are generated by slow cholinergic septal excitation of pyramidal neurons and theta-rhythmic GABAergic inhibition of perisomatic hippocampal interneurons (Buzsáki 2002; Yoder and Pang 2005). This rhythmic inhibition can act as the current source and the excitatory input from the entorhinal cortex to the CA1 pyramidal neuron tuft dendrites as the current sink. In this way, a dipole can emerge that allows for rhythmic current flow from the somata to the dendrites in the extracellular space. This rhythmic current flow can be measured as theta oscillations in the LFP (Buzsáki 2002). The underlying septal rhythmicity might originate in a subpopulation of septal interneurons, which are equipped with ionic channels that promote membrane oscillations and spontaneous firing at theta frequency (Gauss and Seifert 2000; Varga et al. 2008). Theta oscillations accompany voluntary movement, REM sleep and episodes of arousal (Bland and Vanderwolf 1972; Grastyán et al. 1965; Vanderwolf 1971; Whishaw and Vanderwolf 1973). Theta oscillations already occur before the actual theta-associated behavior and thus might contain predictive information about future motor activity. In this way, network excitability could be primed for the routing of information during different behaviors (Fuhrmann et al. 2015; Whishaw and Vanderwolf 1973; Wyble et al. 2004). We have sought to review some of the septo–hippocampal circuit mechanisms and give an overview of the functional implications of the septo–hippocampal connectivity.

## Main

### Cholinergic projections from the medial septum to the hippocampus

The majority of septal projections (~65%) to the hippocampus arise from cholinergic neurons, providing the main source of acetylcholine release in the hippocampus (Sun et al. 2014). In the CA1 sub-region of the hippocampus, the major targets of cholinergic terminals are the proximal dendrites and somata of pyramidal neurons (Frotscher and Léránth 1985; Kiss et al. 1990a, b; Sun et al. 2014; see Fig. 1). The cholinergic neurons in the medial septum fire at low frequencies below 4 Hz in vivo and in vitro and show little or no voltage sag (Simon et al. 2006; Sotty et al. 2003; Zhang et al. 2010). The voltage sag is mediated by the hyperpolarization-activated, cyclic nucleotide-gated non-selective cation channel (HCN) and mediates resonant membrane properties that are thought to facilitate theta rhythmic firing (Hutcheon et al. 1996). The lack of HCN channels might explain why cholinergic medial septal neurons do not show theta rhythmic bursting. The acetylcholine levels in the hippocampus closely follow the low- frequency action potential firing of cholinergic neurons in the medial septum and are elevated specifically in the pyramidal cell layer (Zhang et al. 2010). The cholinergic tone in the hippocampus is generally high during explorative behavior (Day et al. 1991; Stanley et al. 2012; Zhang et al. 2010). The actions of acetylcholine in the hippocampus are complex. Acetylcholine can act via ionotropic nicotinergic receptors and metabotropic muscarinic receptors, which can be expressed pre- or post-synaptically (Cobb and Davies 2005). CA1 pyramidal neurons respond to acetylcholine with membrane potential depolarization, increased input resistance and an elevated spike afterdepolarization. All these actions increase excitability and action potential firing rates (Cole and Nicoll 1984; Dodd et al. 1981; Park and Spruston 2012). The increased intrinsic excitability of CA1 pyramidal neurons, mediated by muscarinic action on A-type potassium channels, leads to a facilitation of dendritic intrinsic plasticity (Losonczy et al. 2008) and facilitates long-term potentiation (Huerta and Lisman 1995; Hyman et al. 2003).

Cholinergic neurons do not show strong phase coupling to hippocampal oscillations but increase their activity during hippocampal theta oscillations (Simon et al. 2006; Zhang et al. 2010). During theta oscillations, the relative power of low-frequency theta is increased by acetylcholine release and the competing non-theta mechanisms are suppressed (Vandecasteele et al. 2014). It is unlikely, however, that septal acetylcholine release during theta oscillations contributes to the extracellular theta currents, since the cholinergic septal neurons fire at low frequencies and the action of acetylcholine on muscarinic receptors is slow. Acetylcholine release might increase the general network excitability rather than setting the pace of theta (Buzsáki 2002). The effects of stimulated acetylcholine release are most prominent in anesthetized animals but are less effective in awake, moving animals. One explanation might be that acetylcholine levels in awake, moving animals are already saturated so that the changes in acetylcholine levels in the hippocampus do not result in a strong modulation of movement-related oscillations (Mamad et al. 2015). During movement of the animal, the contribution of acetylcholine to the generation of theta oscillations may be minor but acetylcholine has been suggested to improve the sensory input-related drive to the hippocampus (Vandecasteele et al. 2014). This is in accordance with the classical view of movement related theta being insensitive to cholinergic antagonists (Kramis et al. 1975).

In CA1 hippocampal interneurons, the activation of muscarinic receptors can mediate membrane depolarization but hyperpolarization and biphasic responses have also been reported (McQuiston 2014). The complexity of cholinergic action on CA1 interneurons is further increased by the highly diverse properties of the CA1 interneuron population, which differ in their firing properties, protein expression and innervation patterns, innervating different compartments of the CA1 pyramidal neurons or other CA1 interneurons. Via the medial septal cholinergic projection, these different interneuron sub-types can be controlled very specifically (McQuiston 2014; Müller and Remy 2014). Due to the different expression patterns of cholinergic receptors on interneurons, different levels of acetylcholine might selectively recruit subsets of interneurons. In this way, a brain state-selective recruitment of interneurons, innervating different layers, could be achieved. The different response-kinetics of nicotinergic and muscarinic cholinergic receptors could also determine the timing of interneuron subtype recruitment. Interneurons that are activated predominantly by muscarinic receptors respond on a slower temporal scale, while interneurons that are activated predominantly by nicotinergic receptors respond faster and more transiently (McQuiston 2014).

In particular, oriens-lacunosum moleculare (O-LM) interneurons, with their somata and dendrites located in stratum oriens and their axonal projections in stratum radiatum and lacunosum moleculare, display fast nicotinergic excitation in response to cholinergic input from the medial septum (Leao et al. 2012). The excitation of this interneuron sub-type is thought to result in a strong inhibition of distal pyramidal neuron dendritic tufts located in stratum lacunosum moleculare, which counteracts the excitation from the entorhinal cortex conveyed via the temporo-ammonic pathway (Fuhrmann et al. 2015; Leao et al. 2012).

In vivo cholinergic septal inputs indeed excite the hippocampal O-LM interneurons sufficiently to cross the action potential threshold (Lovett-Barron et al. 2014). Cholinergic excitation of somatostatin-positive putative O-LM interneurons occurs during the association of multisensory contextual input with an aversive stimulus (Lovett-Barron et al. 2014). But what is their specific role during the association of a multisensory context with an aversive stimulus? When a novel context is learned, the temporo-ammonic pathway conveys multisensory information from the entorhinal cortex to hippocampal pyramidal neuron tuft dendrites (Ahmed and Mehta 2009; Lovett-Barron et al. 2014; Maren and Fanselow 1997). However, when an aversive stimulus occurs in the familiar context, the inputs from the entorhinal cortex coding for the aversive stimulus need to be silenced. Only in this way can the aversive stimulus be associated with the previously learned multisensory context in the amygdala (Fanselow et al. 1993; Lovett-Barron et al. 2014). Somatostatin-positive interneurons, which provide strong inhibition to the CA1 tuft dendrites, are likely candidates to mediate the specific inhibition of temporoammonic excitation; somatostatin-positive interneurons are selectively activated by acetylcholine during the presentation of novel aversive stimuli and their deactivation during fear learning leads to a failure in associating the aversive stimulus with the context (Lovett-Barron et al. 2014). This confirms the hypothesis that O-LM-mediated inhibition of temporo-ammonic excitation via septo–hippocampal acetylcholine release supports fear learning (Lovett-Barron et al. 2014).

To further assess the role of medial septal cholinergic neurons in the behaving animal, several studies employed the immunotoxin saporin conjugated with a cholinergic antibody to selectively lesion cholinergic neurons within the septum. Using specific hippocampus-dependent behavioral tasks, an impairment in the association of places with objects and places with contexts could be observed (Cai et al. 2012; Dannenberg et al. 2016; Easton et al. 2011; Hersman et al. 2017). This again demonstrated that there is a defined role of acetylcholine in the process of associating a unique location with an object or a context. It remains open, however, whether acetylcholine release by medial septal projections to other brain areas might be as relevant.

Furthermore, there is evidence that the cholinergic medial septal input to the hippocampus is important for forming spatial representations in a novel environment (Ikonen et al. 2002). Under control conditions, when an animal was placed from a familiar into a novel environment, the hippocampal place cells changed their spatial representation, a process called remapping (Muller and Kubie 1987; Wilson and McNaughton 1993). In animals with a selective immunotoxic lesion of cholinergic septal neurons projecting to the hippocampus, no novel spatial representations were formed; the place cells retained their firing fields that they had obtained in the familiar environment (Ikonen et al. 2002). Since the neurons’ basic firing properties in a familiar environment were not affected by the specific lesions of cholinergic projections from the medial septum to the hippocampus, a main role of medial septal acetylcholine might be to enable the processing of novel sensory inputs.

### Glutamatergic projections from the medial septum to the hippocampus

Glutamatergic neurons account for approximately 23% of the projections from the medial septum to the hippocampus (Colom et al. 2005). They are characterized by the expression of VGluT1 and/or VGluT2 and by the lack of expression of either ChAT or GAD (Sotty et al. 2003). Electrophysiologically, medial septal glutamatergic neurons form a highly diverse group (Huh et al. 2010; Sotty et al. 2003). The VGluT2 expressing medial septal neurons can be separated into four groups.

The first and largest group is formed by the fast spiking neurons, showing only little action potential accommodation and sometimes spontaneous action potential firing (Huh et al. 2010). Remarkably, some of the fast-spiking glutamatergic neurons show a pronounced sag in response to a hyperpolarizing current injection. Similar intrinsic properties can be observed in GABAergic medial septal neurons (Huh et al. 2010). The second group of VGluT2-positive medial septal neurons exhibit a quite specific firing pattern. These neurons fire clusters of action potentials, which cannot be observed in other cell types of the medial septum. In these neurons, subthreshold intrinsic membrane oscillations, only a small or no sag and strong action potential accommodation is seen. The third group is formed by burst firing glutamatergic neurons, exhibiting a small or no sag (Huh et al. 2010). The neurons of the fourth group are slow firing. Following somatic current injection, they discharge at low rates with accommodating action potentials. The in vivo firing patterns of identified glutamatergic medial septal units are still missing.

Glutamatergic medial septal neurons mainly project to hippocampal interneurons (see Fig. 1) with their somata located in stratum oriens near the alveus. In vivo, the activity of glutamatergic medial septal neurons increases before the mouse initiates locomotion and is higher during running, when compared to resting phases. Not only does the activity of glutamatergic neurons predict the initiation of locomotion but their activity contains further information about the upcoming running episode, as both the firing rates and the number of active glutamatergic neurons reliably predict the future running speed (Fuhrmann et al. 2015). The glutamatergic septo–hippocampal projections terminate on alveus/oriens interneurons in CA1 and activate them in a speed-dependent manner. A large proportion of CA1 alveus/oriens interneurons, including the O-LM cells, are characterized by the expression of somatostatin (Freund and Buzsáki 1996). It has been shown that O-LM interneurons can disinhibit CA1 pyramidal neurons by inhibiting local feed forward interneurons in stratum radiatum and lacunosum moleculare (Fuhrmann et al. 2015; Leao et al. 2012). In this way, the integration of excitatory inputs on pyramidal neurons dendrites is facilitated. This action parallels dendritic inhibition that O-LM interneurons provide onto the distal tuft dendrites of CA1 pyramidal cells in stratum lacunosum-moleculare. In this way, somatostatin-positive interneurons, which can be activated by cholinergic (see also “Cholinergic projections from the medial septum to the hippocampus") or glutamatergic septal input, might have a net inhibitory effect on distal CA1 pyramidal neuron dendrites (via O-LM-mediated dendritic inhibition; Lovett-Barron et al. 2014; Maccaferri and McBain 1995) and a net disinhibitory effect onto proximal dendrites (via reduction of feed forward inhibition; Fuhrmann et al. 2015; Leao et al. 2012). Somatostatin is expressed by several cell types with their somata located in stratum oriens (Bezaire and Soltesz 2013; Freund and Buzsáki 1996). O-LM cells represent a non-uniform subpopulation of somatostatin-expressing cells (Mikulovic et al. 2015). Whether the somatostatin-positive interneurons, recruited during different behavioral tasks by glutamatergic or cholinergic septal innervation, represent a uniform population or different neuronal sub-classes is an interesting open question.

Both the CA1 pyramidal neuron population and alveus/oriens interneurons show increased firing rates when activated by glutamatergic septo–hippocampal projections at higher running speeds (Fuhrmann et al. 2015). Mechanistically, this is likely achieved by a facilitation of input summation onto CA1 dendrites via disinhibition. As a result, CA1 network excitability can be tuned by glutamatergic projections from the septum via a dynamic modulation of excitatory and inhibitory microcircuits in a locomotion speed-dependent manner. This circuit may be differentially employed by different medial septal activation patterns during certain behavioral states (Simon et al. 2006). Interestingly, the behavioral state transition is signaled hundreds of milliseconds before the initiation of motor activity (Fuhrmann et al. 2015), so that medial septal glutamatergic neurons already shift the CA1 network to a higher excitability before the onset of a running episode. Thus, the medial septum may serve to prime the hippocampal network for processing of environmental and spatial inputs during translational movement (Fuhrmann et al. 2015).

It is tempting to speculate that behavioral state-dependent regulation of hippocampal inhibition influences the process of place field formation of CA1 principal cells. There is strong experimental evidence that dendritic nonlinear events, plateau potentials, are mechanistically involved in place field formation (Bittner et al. 2015). Initiation of plateau potentials and other non-linear dendritic events have been shown to be under strong inhibitory control (Grienberger et al. 2017; Müller et al. 2012). Thus, dendritic non-linear events and concomitant plasticity might be facilitated at higher locomotion speeds through reduced inhibition. For spatial coding, it has been shown that inhibition suppresses out-of-field excitation, which increases place field precision (Grienberger et al. 2017). Decreased inhibition correlating with increased running speeds could trade the spatial precision of place cell output for an increased output probability. By allowing more out-of-field excitation to evoke output, the spatial tuning might be less precise but the output reliability in a place field could be increased.

Recent work on the role of glutamatergic neurons in the medial septum provides new insight into the cellular mechanisms underlying movement-associated theta oscillations (Fuhrmann et al. 2015). In these experiments, the optogenetic activation of glutamatergic septal neurons in the theta frequency band led to an entrainment of stimulus-frequency locked LFP oscillations in CA1 (Fuhrmann et al. 2015; Robinson et al. 2016). Following the induction of hippocampal theta oscillations by a rhythmic stimulation of glutamatergic septal neurons, locomotion was initiated within several hundreds of milliseconds (Fuhrmann et al. 2015). The higher the frequency of the stimulated theta, the shorter the time lag between the stimulation and the resulting running initiation and the higher the subsequent running speed. Even a short stimulation of septal glutamatergic neurons below 1 s could entrain self-sustaining hippocampal theta with subsequent running initiation (Fuhrmann et al. 2015). Also, when running was initiated spontaneously, movement-associated theta increased in amplitude and the theta frequency increased in correlation with the upcoming running speed (Li et al. 2012; Rivas et al. 1996). Interestingly, in experiments in which animals had to jump to different heights for shock avoidance, theta frequency increased with increasing heights that had to be reached by the jump (Morris et al. 1976; Whishaw and Vanderwolf 1973). These findings show that the predictive motif of theta also applies to movement types other than running. Thus, theta oscillations might more generally predict the vigor of the intended movement (Vanderwolf 1969; Wyble et al. 2004).

Pharmacological blockade of local glutamatergic transmission to cholinergic and GABAergic neurons locally in the medial septum strongly reduced hippocampal theta oscillations (Fuhrmann et al. 2015). However, locomotion could still be induced by stimulating the glutamatergic medial septal neurons and spontaneous locomotion could also still be observed (Fuhrmann et al. 2015). The most likely explanation for this observation is that the intra-septal glutamatergic activation of non-glutamatergic neurons is required for hippocampal theta generation. Furthermore, it can be concluded that the induction of locomotor activity is a direct glutamatergic effect of septo–fugal projections. Remarkably, during the intra-septal blockade of glutamatergic transmission, the correlation between theta frequency and locomotion velocity was strongly reduced (Fuhrmann et al. 2015; Robinson et al. 2016). This implies that the intra-septal glutamatergic activation of non-glutamatergic neurons in the septum is involved in the coupling of hippocampal theta frequency to the running velocity.

Not only stimulation of glutamatergic medial septal neurons has been shown to induce locomotor activity but also the electrical stimulation of the posterior hypothalamus, which provides input to the medial septum, effectively triggering locomotor activity (Bland and Oddie 2001). Pharmacological silencing of the medial septum during hypothalamic stimulation leads to a reduction of both hippocampal theta oscillations and locomotor activation. This suggests that the coupling of theta oscillations and movement might indeed occur on the level of the medial septum (Oddie et al. 1996). Furthermore, there is strong evidence for subcortical modulation of the medial septum by afferents from the median raphe nucleus, the locus coeruleus and other hypothalamic sub-regions (Carter et al. 2010; Fuhrmann et al. 2015; Moore 1978; Vertes 1988). It remains to be shown, however, if the effects mediated by these afferent regions onto the septal activity influences theta oscillations, movement initiation, or both. Undoubtedly, there is increasing evidence that information about the locomotor state and the running speed is provided by septo–hippocampal and septo–entorhinal projections to neurons that are involved in encoding space (Fuhrmann et al. 2015; Justus et al. 2016).

### GABAergic projections from the medial septum to the hippocampus

GABAergic neurons in the medial septum are a non-uniform group. They can be distinguished with respect to the expression patterns of the calcium-binding protein parvalbumin (PV), the neuropeptide somatostatin and the presence of cyclic nucleotide gated hyperpolarization activated ion channels (HCN; Freund 1989; Sotty et al. 2003; Varga et al. 2008). Medial septal parvalbumin-positive GABAergic neurons have been found to generally discharge at higher frequencies than parvalbumin-negative GABAergic neurons within the medial septum (Simon et al. 2006). In response to long current injections in brain slices, GABAergic septal neurons show characteristic fast-spiking or burst-firing behavior (Sotty et al. 2003). In contrast to cholinergic septal neurons, GABAergic neurons display theta-coupled burst firing in vivo. The theta rhythmic firing of the septal GABAergic neurons is tightly coupled to the trough or the peak of theta (Borhegyi 2004). A subpopulation of the parvalbumin-positive neurons in the medial septum expresses HCN channels and fire tightly coupled to hippocampal theta oscillations (Varga et al. 2008). Through strong local intra-septal connectivity, the GABAergic medial septal neurons have been found to mediate theta synchronization of the local network (Borhegyi 2004). This theta rhythmicity is then transmitted via septo–hippocampal projections to the hippocampus. The sub-group of GABAergic septal neurons, expressing HCN and parvalbumin, are likely candidates to provide this theta rhythmic drive to the hippocampus (Varga et al. 2008).

GABAergic projections from the medial septum predominately target hippocampal GABAergic interneurons expressing parvalbumin (Freund 1989; Freund and Antal 1988; Sun et al. 2014). One main role of parvalbumin positive hippocampal interneurons, in particular of the parvalbumin-positive basket cells, is to provide powerful synchronous inhibition to the perisomatic region of CA1 pyramidal neurons (Freund and Katona 2007). In this way, the rhythmic activation of medial septal GABAergic neurons might be transformed into rhythmic disinhibition of the hippocampal pyramidal neuron population and a synchronization between hippocampal and medial septal networks can be achieved (Alonso and Köhler 1982; Hangya et al. 2009; Toth et al. 1997). The theta rhythmic firing of PV/HCN-positive GABAergic neurons in the medial septum precedes the rhythmic discharge of putative GABAergic neurons in the hippocampus (Hangya et al. 2009). This rhythmic activation of the local GABAergic interneurons in the hippocampus precedes the local field potential (Hangya et al. 2009). Optogenetic activation of the septo–hippocampal GABAergic neurons increases hippocampal theta oscillations, whereas optogenetic silencing of these neurons strongly reduces hippocampal theta (Bender et al. 2015; Boyce et al. 2016; Gangadharan et al. 2016). These observations strongly support the notion that septal GABAergic projections mediate the hippocampal field potential oscillations via theta rhythmic activation of hippocampal interneurons (Buzsáki 2002). In marked contrast to the increased rhythmic firing during theta oscillations, GABAergic neurons in the medial septum are suppressed during other brain states, for example during hippocampal sharp-wave ripples (Dragoi et al. 1999). This implies a different functional coupling of septal GABAergic neurons to the local hippocampal network in a brain state-dependent manner.

The input strength from putative GABAergic septal neurons to hippocampal interneurons as well as the theta power increases during running episodes (Kaifosh et al. 2013). This finding is in agreement with the fact that GABAergic septal input to hippocampal interneurons is highly correlated to hippocampal theta oscillations. In addition, the presentation of different sensory stimuli results in an activation of these GABAergic septal inputs onto hippocampal interneurons (Kaifosh et al. 2013). This activation of septo–hippocampal GABAergic projection neurons increases with sensory stimulus intensity, irrelevant of the modality of the sensory input. Both during running episodes and when sensory stimuli are presented the input from medial septal GABAergic neurons to hippocampal interneurons increases in strength; theta oscillations only increase in power during locomotion (Kaifosh et al. 2013). This suggests that the theta generation may not be exclusively controlled by medial septal GABAergic projections (Kaifosh et al. 2013). There is evidence that the direct input from brain stem and hypothalamic nuclei provides sensory information (Kaifosh et al. 2013). In this way, the excitability of the hippocampal network could be adjusted by sensory inputs from subcortical and cortical areas via septal GABAergic projections. The initiation and entrainment of theta during running episodes could be provided by a different circuit, e.g., the glutamatergic intra-septal circuitry that may recruit local medial septal GABAergic neurons and their septo–hippocampal projections (see “Glutamatergic projections from the medial septum to the hippocampus”).

### GABAergic projections from the hippocampus to the medial septum

GABAergic neurons in the hippocampus not only receive strong GABAergic input from the medial septum but they can also project back to the medial septum (Alonso and Köhler 1982; Takács et al. 2008; Tóth et al. 1993). In this way, they form a reciprocal long-range GABAergic septo-hippocampal circuit. Many long-range GABAergic neurons simultaneously form local synapses in CA1 and en passant synapses in several remote areas (Gulyás et al. 2003; Takács et al. 2008). The long-range projecting axons of the GABAergic neurons are highly myelinated, which argues for a specific role in the immediate synchronization and functional binding of remote areas (Caputi et al. 2013).

The GABAergic neurons projecting from the medial septum to the hippocampus are predominantly parvalbumin positive. In contrast, the GABAergic neurons projecting from the hippocampus to the septum are predominantly somtatostatin-expressing neurons (Jinno and Kosaka 2002). The hippocampal GABAergic projection neurons mainly target parvalbumin-expressing GABAergic neurons and to a lesser amount cholinergic neurons in the medial septum (Tóth et al. 1993). Input from hippocampal GABAergic neurons mediates most likely a fast inhibitory response in medial septal GABAergic neurons and a slow inhibitory response in medial septal cholinergic neurons (Mattis et al. 2014). GABAergic neurons in the hippocampus, which project to the medial septum, are located in the stratum oriens of the hippocampus, the layer in which the majority of the septo–hippocampal projections terminates (Jinno et al. 2007). And, indeed, GABAergic medial septal neurons have been identified to project to the same GABAergic neuron in the hippocampus, from which they receive input (Takács et al. 2008). This demonstrates a direct reciprocity within the septo–hippocampal GABAergic network.

In vivo long-range GABAergic neurons in the hippocampus display rhythmic firing; however, they are not forming a uniform group regarding their discharge patterns (Katona et al. 2017). During sharp-wave ripples, most neurons in CA1 increase their firing rates (Csicsvari et al. 1999), which is thought to result from strong excitatory input from CA3. This strong activity in the hippocampus appears not to be transmitted via long-range projecting GABAergic neurons to the medial septum, so that no increased activity in the medial septum can be observed during sharp-waves. In contrast, theta rhythmic activity is conveyed between the hippocampus and the medial septum in both directions. This indicates that the reciprocal connection between the hippocampus and the medial septum possesses different functions depending on the behavioral state (Dragoi et al. 1999).

## Conclusion

**Fig. 2:**
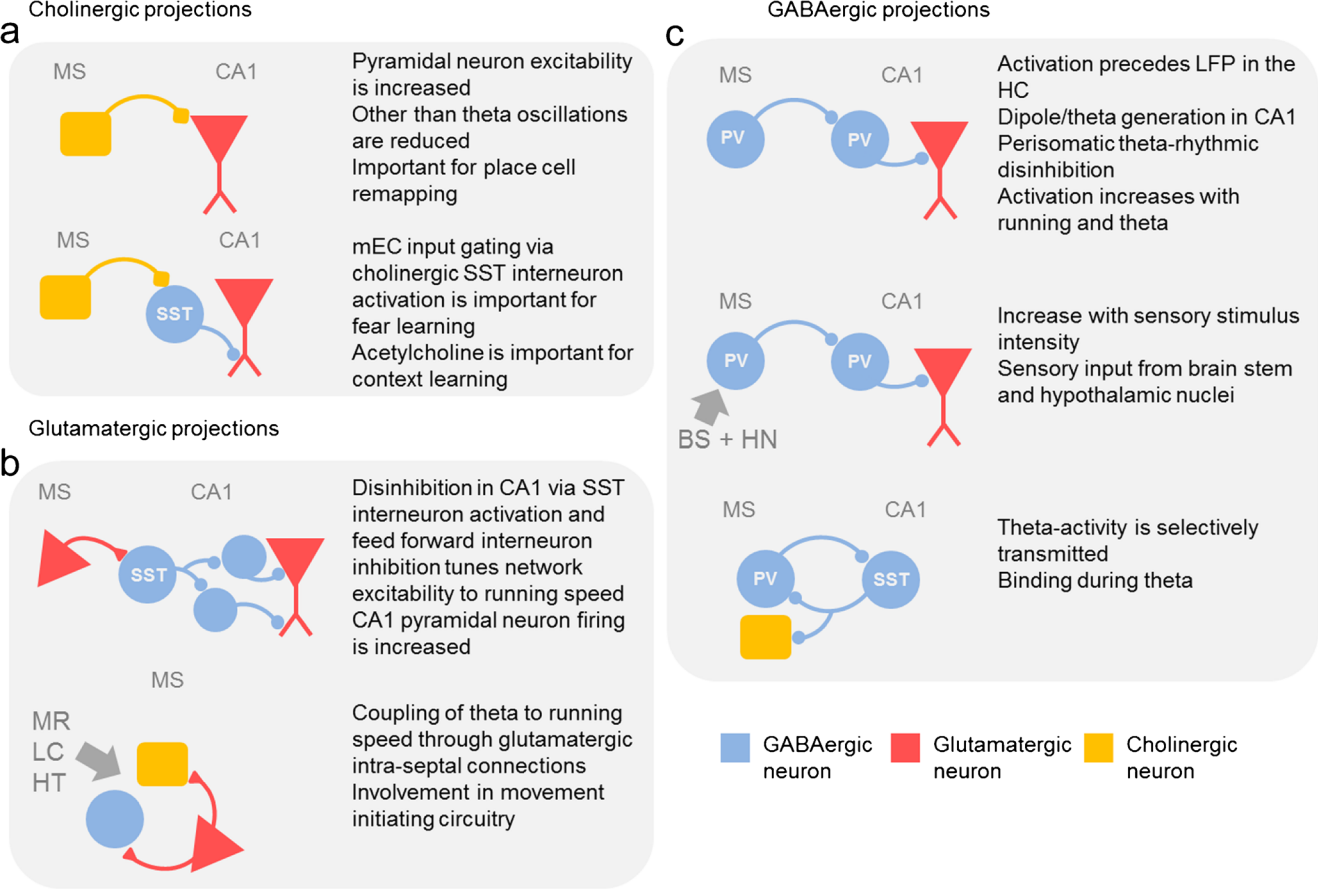
Septo-hippocampal network interactions.** a** Schematic drawing of cholinergic connections between the medial septum (*MS*) and the hippocampal CA1 sub-region and their summarized functional implications.* SST* somatostatin positive GABAergic neurons. **b** Schematic drawing of glutamatergic connections between the medial septum (*MS*) and the hippocampal CA1 sub-region, intra-septal glutamatergic connections and their summarized functional implications.* MR* median raphe nucleus,* LC* locus coeruleus.* HT* hypothalamus. **c** Schematic drawing of GABAergic connections between the medial septum (*MS*) and the hippocampal CA1 sub-region and vice versa and their summarized functional implications.* BS* brain stem nuclei,* HN* hypothalamic nuclei,* PV* parvalbumin-positive GABAergic neurons

In this review, on septo–hippocampal interaction that by far could not cover the full extent of the literature, we pointed out the properties and specific roles of major medial septal cell types and their projections. Cholinergic medial septal neurons do not couple to theta oscillations but their firing rates during theta oscillations are elevated. Acetylcholine is thought to suppress oscillations in other frequencies than theta and is released during exploration and associative learning tasks (Vandecasteele et al. 2014). This increases the intrinsic excitability of pyramidal neurons in the hippocampus, thus increasing their responses to certain inputs. By activating somatostatin-positive interneurons in stratum oriens, cholinergic septal input might control the information flow transmitted via layered input onto proximal and apical tuft dendrites of CA1 pyramidal neurons. Cholinergic input is also involved in the process of hippocampal place cell remapping in novel environments. Thus, the cholinergic septo–hippocampal connections may be functionally involved in the differential tuning of the pyramidal neuron excitability in novel and familiar environments (Cohen et al. 2017; see Fig. 2a).

Glutamatergic septal neurons activate hippocampal interneurons in stratum oriens before and during movement. Their activity rates are elevated during locomotion and correlated to the animal’s running velocity. Thereby, they provide a speed signal to the CA1 pyramidal neurons and may serve to adjust hippocampal excitability to the vigor of future and ongoing locomotor activity. The local glutamatergic network within the medial septum may provide the coupling of hippocampal theta oscillations to the running velocity. The medial septum is located in a central position in the locomotion-initiation circuitry and is well interconnected with subcortical regions on the input and output level (see Fig. 2b).

GABAergic septal projections to the hippocampus predominantly terminate on GABAergic parvalbumin positive neurons in the hippocampus. They synchronize and entrain the local inhibitory, mainly perisomatically innervating interneuron population to the theta rhythm. In this way, they rhythmically disinhibit the pyramidal neuron population and are main contributors to theta generation in the hippocampus. GABAergic input from the medial septum furthermore carries information about the intensity of sensory stimuli. The reciprocity in the GABAergic connection between the hippocampus and the medial septum may serve to ensure the binding and synchrony of both brain areas in a brain state-dependent manner (see Fig. 2c).

Neurons in the medial septum innervate hippocampal pyramidal neurons to adjust their excitability directly. Furthermore, a variety of CA1 interneurons is targeted to orchestrate the hippocampal network activity in many facets. The diversity of innervation patterns, time-courses of activation and rhythmic firing properties of these interneurons makes them perfect relay stations for fine-tuning the hippocampal network excitability during changes of the behavioral state. Inhibitory projections from the medial septum target mostly the PV-positive hippocampal interneurons for rhythmogenesis. Excitatory septo–hippocampal projections target the group of somatostatin-positive stratum oriens interneurons, including the O-LM interneurons. The medial septum has at least two ways to provide excitation to these neurons, first via glutamatergic and second, via cholinergic, projections; somatostatin-positive interneurons stand out for several reasons: Somatostatin-positive interneurons can project to all hippocampal layers and thereby control the excitation from the temporo-ammonic and the Schaffer collateral pathway (Fuhrmann et al. 2015, Leao et al. 2012). In this way, the medial septum may route inputs to the hippocampus via pathway-dependent disinhibition. Hippocampal GABAergic neurons projecting from the hippocampus to the medial septum are also somatostatin-positive (Gulyás et al. 2003). Thus, somatostatin-positive neurons in the hippocampus might be allocated with a central position to mediate the interaction of the medial septal and the hippocampal network.

## References

[CR1] Ahmed OJ, Mehta MR (2009). The hippocampal rate code: anatomy, physiology and theory. Trends Neurosci.

[CR2] Alonso A, Köhler C (1982). Evidence for separate projections of hippocampal pyramidal and non-pyramidal neurons to different parts of the septum in the rat brain. Neurosci Lett.

[CR3] Amaral DG, Witter MP (1989). The three-dimensional organization of the hippocampal formation: a review of anatomical data. Neuroscience.

[CR4] Anderson, P., R. Morris, D. Amaral, T. Bliss, and J. O’Keefe (Eds.) (2007) The Hippocampus Book. Oxford University Press, Oxford

[CR5] Bender F, Gorbati M, Cadavieco MC, Denisova N, Gao X, Holman C, Korotkova T, Ponomarenko A (2015). Theta oscillations regulate the speed of locomotion via a hippocampus to lateral septum pathway. Nat Commun.

[CR6] Bezaire MJ, Soltesz I (2013). Quantitative assessment of CA1 local circuits: knowledge base for interneuron-pyramidal cell connectivity. Hippocampus.

[CR7] Bittner KC, Grienberger C, Vaidya SP, Milstein AD, Macklin JJ, Suh J, Tonegawa S, Magee JC (2015). Conjunctive input processing drives feature selectivity in hippocampal ca1 neurons. Nat Neurosci.

[CR8] Bland BH, Oddie SD (2001). Theta band oscillation and synchrony in the hippocampal formation and associated structures: the case for its role in sensorimotor integration. Behav Brain Res.

[CR9] Bland BH, Vanderwolf CH (1972). Electrical stimulation of the hippocampal formation: behavioral and bioelectrical effects. Brain Res.

[CR10] Borhegyi Z (2004) Phase segregation of medial septal gabaergic neurons during hippocampal theta activity. J Neurosci 24(39):8470–8479 10.1523/JNEUROSCI.1413-04.2004 PMC6729892 15456820

[CR11] Boyce R, Glasgow SD, Williams S, Adamantidis A (2016). Causal evidence for the role of rem sleep theta rhythm in contextual memory consolidation. Science.

[CR12] Buzsáki G (2002). Theta oscillations in the hippocampus. Neuron.

[CR13] Cai L, Gibbs RB, Johnson DA (2012). Recognition of novel objects and their location in rats with selective cholinergic lesion of the medial septum. Neurosci Lett.

[CR14] Caputi A, Melzer S, Michael M, Monyer H (2013). The long and short of gabaergic neurons. Curr Opin Neurobiol.

[CR15] Carter ME, Yizhar O, Chikahisa S, Nguyen H, Adamantidis A, Nishino S, Deisseroth K, de Lecea L (2010). Tuning arousal with optogenetic modulation of locus coeruleus neurons. Nat Neurosci.

[CR16] Cobb SR, Davies CH (2005). Cholinergic modulation of hippocampal cells and circuits. J Physiol.

[CR17] Cohen, J. D., M. Bolstad, A. K. Lee (2017) Experience-dependent shaping of hippocampal CA1 intracellular activity in novel and familiar environments. eLife 6:e23040 10.7554/eLife.23040 PMC5526666 28742496

[CR18] Cole AE, Nicoll RA (1984). The pharmacology of cholinergic excitatory responses in hippocampal pyramidal cells. Brain Res.

[CR19] Colom LV, Castaneda MT, Reyna T, Hernandez S, Garrido-Sanabria E (2005). Characterization of medial septal glutamatergic neurons and their projection to the hippocampus. Synapse.

[CR20] Csicsvari J, Hirase H, Czurkó A, Mamiya A, Buzsáki G (1999). Oscillatory coupling of hippocampal pyramidal cells and interneurons in the behaving rat. J Neurosci.

[CR21] Dannenberg H, Hinman JR, Hasselmo ME (2016). Potential roles of cholinergic modulation in the neural coding of location and movement speed. J Physiol Paris.

[CR22] Day J, Damsma G, Fibiger HC (1991). Cholinergic activity in the rat hippocampus, cortex and striatum correlates with locomotor activity: an in vivo microdialysis study. Pharmacol Biochem Behav.

[CR23] Dodd J, Dingledine R, Kelly JS (1981). The excitatory action of acetylcholine on hippocampal neurones of the guinea pig and rat maintained in vitro. Brain Res.

[CR24] Dragoi G, Carpi D, Recce M, Csicsvari J, Buzsáki G (1999). Interactions between hippocampus and medial septum during sharp waves and theta oscillation in the behaving rat. J Neurosci.

[CR25] Easton A, Fitchett AE, Eacott MJ, Baxter MG (2011). Medial septal cholinergic neurons are necessary for context-place memory but not episodic-like memory. Hippocampus.

[CR26] Eichenbaum H (2017). Prefrontal-hippocampal interactions in episodic memory. Nat Rev Neurosci.

[CR27] Fanselow MS, DeCola JP, Young SL (1993). Mechanisms responsible for reduced contextual conditioning with massed unsignaled unconditional stimuli. J Exp Psychol Anim Behav Process.

[CR28] Freund TF (1989). Gabaergic septohippocampal neurons contain parvalbumin. Brain Res.

[CR29] Freund TF, Antal M (1988). GABA-containing neurons in the septum control inhibitory interneurons in the hippocampus. Nature.

[CR30] Freund TF, Buzsáki G (1996). Interneurons of the hippocampus. Hippocampus.

[CR31] Freund TF, Katona I (2007). Perisomatic inhibition. Neuron.

[CR32] Frotscher M, Léránth C (1985). Cholinergic innervation of the rat hippocampus as revealed by choline acetyltransferase immunocytochemistry: a combined light and electron microscopic study. J Comp Neurol.

[CR33] Fuhrmann F, Justus D, Sosulina L, Kaneko H, Beutel T, Friedrichs D, Schoch S, Schwarz MK, Fuhrmann M, Remy S (2015). Locomotion, theta oscillations, and the speed-correlated firing of hippocampal neurons are controlled by a medial septal glutamatergic circuit. Neuron.

[CR34] Gangadharan G, Shin J, Kim S-W, Kim A, Paydar A, Kim D-S, Miyazaki T, Watanabe M, Yanagawa Y, Kim J, Kim Y-S, Kim D, Shin H-S (2016). Medial septal gabaergic projection neurons promote object exploration behavior and type 2 theta rhythm. Proc Natl Acad Sci U S A.

[CR35] Gauss R, Seifert R (2000). Pacemaker oscillations in heart and brain: a key role for hyperpolarization-activated cation channels. Chronobiol Int.

[CR36] Grastyán E, Karmos G, Vereczkey L, Martin J, Kellenyi L (1965). Hypothalamic motivational processes as reflected by their hippocampal electrical correlates. Science.

[CR37] Grienberger, C., A. D. Milstein, K. C. Bittner, S. Romani, and J. C. Magee (2017) Inhibitory suppression of heterogeneously tuned excitation enhances spatial coding in ca1 place cells. Nat Neurosci 20: 417–426 10.1038/nn.4486 28114296

[CR38] Gulyás AI, Hájos N, Katona I, Freund TF (2003). Interneurons are the local targets of hippocampal inhibitory cells which project to the medial septum. Eur J Neurosci.

[CR39] Hajszan T, Alreja M, Leranth C (2004). Intrinsic vesicular glutamate transporter 2-immunoreactive input to septohippocampal parvalbumin-containing neurons: novel glutamatergic local circuit cells. Hippocampus.

[CR40] Hangya B, Borhegyi Z, Szilágyi N, Freund TF, Varga V (2009). Gabaergic neurons of the medial septum lead the hippocampal network during theta activity. J Neurosci.

[CR41] Hersman S, Cushman J, Lemelson N, Wassum K, Lotfipour S, Fanselow MS (2017). Optogenetic excitation of cholinergic inputs to hippocampus primes future contextual fear associations. Sci Rep.

[CR42] Huerta PT, Lisman JE (1995). Bidirectional synaptic plasticity induced by a single burst during cholinergic theta oscillation in ca1 in vitro. Neuron.

[CR43] Huh CYL, Goutagny R, Williams S (2010). Glutamatergic neurons of the mouse medial septum and diagonal band of broca synaptically drive hippocampal pyramidal cells: relevance for hippocampal theta rhythm. J Neurosci.

[CR44] Hutcheon B, Miura RM, Puil E (1996). Subthreshold membrane resonance in neocortical neurons. J Neurophysiol.

[CR45] Hyman JM, Wyble BP, Goyal V, Rossi CA, Hasselmo ME (2003). Stimulation in hippocampal region ca1 in behaving rats yields long-term potentiation when delivered to the peak of theta and long-term depression when delivered to the trough. J Neurosci.

[CR46] Ikonen S, McMahan R, Gallagher M, Eichenbaum H, Tanila H (2002). Cholinergic system regulation of spatial representation by the hippocampus. Hippocampus.

[CR47] Jinno S, Kosaka T (2002). Immunocytochemical characterization of hippocamposeptal projecting gabaergic nonprincipal neurons in the mouse brain: a retrograde labeling study. Brain Res.

[CR48] Jinno S, Klausberger T, Marton LF, Dalezios Y, Roberts JDB, Fuentealba P, Bushong EA, Henze D, Buzsáki G, Somogyi P (2007). Neuronal diversity in gabaergic long-range projections from the hippocampus. J Neurosci.

[CR49] Justus, D., D. Dalügge, S. Bothe, F. Fuhrmann, C. Hannes, H. Kaneko, D. Friedrichs, L. Sosulina, I. Schwarz, D. A. Elliott et al. (2016) Glutamatergic synaptic integration of locomotion speed via septoentorhinal projections. Nat Neurosci 20, 16–19 10.1038/nn.4447 27893726

[CR50] Kaifosh P, Lovett-Barron M, Turi GF, Reardon TR, Losonczy A (2013). Septo-hippocampal GABAergic signaling across multiple modalities in awake mice. Nat Neurosci.

[CR51] Katona L, Micklem B, Borhegyi Z, Swiejkowski DA, Valenti O, Viney TJ, Kotzadimitriou D, Klausberger T, Somogyi P (2017). Behavior-dependent activity patterns of gabaergic long-range projecting neurons in the rat hippocampus. Hippocampus.

[CR52] King C, Recce M, O’Keefe J (1998). The rhythmicity of cells of the medial septum/diagonal band of broca in the awake freely moving rat: relationships with behaviour and hippocampal theta. Eur J Neurosci.

[CR53] Kiss J, Patel AJ, Baimbridge KG, Freund TF (1990). Topographical localization of neurons containing parvalbumin and choline acetyltransferase in the medial septum-diagonal band region of the rat. Neuroscience.

[CR54] Kiss J, Patel AJ, Freund TF (1990). Distribution of septohippocampal neurons containing parvalbumin or choline acetyltransferase in the rat brain. J Comp Neurol.

[CR55] Kramis R, Vanderwolf CH, Bland BH (1975). Two types of hippocampal rhythmical slow activity in both the rabbit and the rat: relations to behavior and effects of atropine, diethyl ether, urethane, and pentobarbital. Exp Neurol.

[CR56] Leao RN, Mikulovic S, Leao KE, Munguba H, Gezelius H, Enjin A, Patra K, Eriksson A, Loew LM, Tort ABL, Kullander K (2012). OLM interneurons differentially modulate CA3 and entorhinal inputs to hippocampal CA1 neurons. Nat Neurosci.

[CR57] Leao RN, Targino ZH, Colom LV, Fisahn A (2014). Interconnection and synchronization of neuronal populations in the mouse medial septum/diagonal band of broca. J Neurophysiol.

[CR58] Leutgeb S, Leutgeb JK (2007). Pattern separation, pattern completion, and new neuronal codes within a continuous ca3 map. Learn Mem.

[CR59] Li J-Y, Kuo TBJ, Hsieh I-T, Yang CCH (2012). Changes in hippocampal theta rhythm and their correlations with speed during different phases of voluntary wheel running in rats. Neuroscience.

[CR60] Losonczy A, Makara JK, Magee JC (2008). Compartmentalized dendritic plasticity and input feature storage in neurons. Nature.

[CR61] Lovett-Barron M, Kaifosh P, Kheirbek MA, Danielson N, Zaremba JD, Reardon TR, Turi GF, Hen R, Zemelman BV, Losonczy A (2014). Dendritic inhibition in the hippocampus supports fear learning. Science.

[CR62] Maccaferri G, McBain CJ (1995). Passive propagation of LTD to stratum oriens-alveus inhibitory neurons modulates the temporoammonic input to the hippocampal CA1 region. Neuron.

[CR63] Mamad O, McNamara HM, Reilly RB, Tsanov M (2015). Medial septum regulates the hippocampal spatial representation. Front Behav Neurosci.

[CR64] Manseau F, Danik M, Williams S (2005). A functional glutamatergic neurone network in the medial septum and diagonal band area. J Physiol.

[CR65] Maren S, Fanselow MS (1997). Electrolytic lesions of the fimbria/fornix, dorsal hippocampus, or entorhinal cortex produce anterograde deficits in contextual fear conditioning in rats. Neurobiol Learn Mem.

[CR66] Mattis J, Brill J, Evans S, Lerner TN, Davidson TJ, Hyun M, Ramakrishnan C, Deisseroth K, Huguenard JR (2014). Frequency-dependent, cell type-divergent signaling in the hippocamposeptal projection. J Neurosci.

[CR67] McQuiston A R (2014) Acetylcholine release and inhibitory interneuron activity in hippocampal CA1. Front Synaptic Neurosci 6 10.3389/fnsyn.2014.00020 PMC4165287 25278874

[CR68] Mikulovic S, Restrepo CE, Hilscher MM, Kullander K, Leão RN (2015). Novel markers for olm interneurons in the hippocampus. Front Cell Neurosci.

[CR69] Moore RY (1978). Catecholamin innervation of the basal forebrain. i. The septal area. J Comp Neurol.

[CR70] Morris R, Black A, O’Keefe J (1976). Hippocampal eeg during a ballistic movement.

[CR71] Muller RU, Kubie JL (1987). The effects of changes in the environment on the spatial firing of hippocampal complex-spike cells. J Neurosci.

[CR72] Müller C, Remy S (2014). Dendritic inhibition mediated by o-lm and bistratified interneurons in the hippocampus. Front Synaptic Neurosci.

[CR73] Müller C, Beck H, Coulter D, Remy S (2012) Inhibitory control of linear and supralinear dendritic excitation in CA1 pyramidal neurons. Neuron 75(5):851–864 10.1016/j.neuron.2012.06.025 22958825

[CR74] O’Keefe (1979) A review of the hippocampal place cells. Prog Neurobiol 13:419–439 10.1016/0301-0082(79)90005-4 396576

[CR75] O’Keefe J, Recce ML (1993). Phase relationship between hippocampal place units and the EEG theta rhythm. Hippocampus.

[CR76] Oddie SD, Stefanek W, Kirk IJ, Bland BH (1996). Intraseptal procaine abolishes hypothalamic stimulation-induced wheel-running and hippocampal theta field activity in rats. J Neurosci.

[CR77] Park J-Y, Spruston N (2012). Synergistic actions of metabotropic acetylcholine and glutamate receptors on the excitability of hippocampal ca1 pyramidal neurons. J Neurosci.

[CR78] Rivas J, Gaztelu JM, Garcia-Austt E (1996). Changes in hippocampal cell discharge patterns and theta rhythm spectral properties as a function of walking velocity in the guinea pig. Exp Brain Res.

[CR79] Robinson J, Manseau F, Ducharme G, Amilhon B, Vigneault E, El Mestikawy S, Williams S (2016). Optogenetic activation of septal glutamatergic neurons drive hippocampal theta rhythms. J Neurosci.

[CR80] Simon AP, Poindessous-Jazat F, Dutar P, Epelbaum J, Bassant M-H (2006). Firing properties of anatomically identified neurons in the medial septum of anesthetized and unanesthetized restrained rats. J Neurosci.

[CR81] Sotty F, Danik M, Manseau F, Laplante F, Quirion R, Williams S (2003). Distinct electrophysiological properties of glutamatergic, cholinergic and gabaergic rat septohippocampal neurons: novel implications for hippocampal rhythmicity. J Physiol.

[CR82] Stanley EM, Wilson MA, Fadel JR (2012). Hippocampal neurotransmitter efflux during one-trial novel object recognition in rats. Neurosci Lett.

[CR83] Sun Y, Nguyen AQ, Nguyen JP, Le L, Saur D, Choi J, Callaway EM, Xu X (2014). Cell-type-specific circuit connectivity of hippocampal CA1 revealed through cre-dependent rabies tracing. Cell Rep.

[CR84] Swanson LW, Cowan WM (1979). The connections of the septal region in the rat. J Comp Neurol.

[CR85] Takács VT, Freund TF, Gulyás AI (2008). Types and synaptic connections of hippocampal inhibitory neurons reciprocally connected with the medial septum. Eur J Neurosci.

[CR86] Tóth K, Borhegyi Z, Freund TF (1993). Postsynaptic targets of gabaergic hippocampal neurons in the medial septum-diagonal band of broca complex. J Neurosci.

[CR87] Toth K, Freund TF, Miles R (1997). Disinhibition of rat hippocampal pyramidal cells by gabaergic afferents from the septum. J Physiol.

[CR88] Vandecasteele, M., V. Varga, A. Berényi, E. Papp, P. Barthó, L. Venance, T. F. Freund, G. Buzsáki (2014) Optogenetic activation of septal cholinergic neurons suppresses sharp wave ripples and enhances theta oscillations in the hippocampus. Proc Natl Acad Sci U S A 111:13535–13540 10.1073/pnas.1411233111 PMC4169920 25197052

[CR89] Vanderwolf, C H (1969) Hippocampal electrical activity and voluntary movement in the rat. Electroencephalogr Clin Neurophysiol 26(4): 407–418 10.1016/0013-4694(69)90092-3 4183562

[CR90] Vanderwolf CH (1971). Limbic-diencephalic mechanisms of voluntary movement. Psychol Rev.

[CR91] Varga V, Hangya B, Kránitz K, Ludányi A, Zemankovics R, Katona I, Shigemoto R, Freund TF, Borhegyi Z (2008). The presence of pacemaker hcn channels identifies theta rhythmic gabaergic neurons in the medial septum. J Physiol.

[CR92] Vertes RP (1988). Brainstem afferents to the basal forebrain in the rat. Neuroscience.

[CR93] Whishaw IQ, Vanderwolf CH (1973). Hippocampal eeg and behavior: changes in amplitude and frequency of rsa (theta rhythm) associated with spontaneous and learned movement patterns in rats and cats. Behav Biol.

[CR94] Wilson MA, McNaughton BL (1993). Dynamics of the hippocampal ensemble code for space. Science.

[CR95] Wyble BP, Hyman JM, Rossi CA, Hasselmo ME (2004). Analysis of theta power in hippocampal eeg during bar pressing and running behavior in rats during distinct behavioral contexts. Hippocampus.

[CR96] Yoder RM, Pang KCH (2005). Involvement of gabaergic and cholinergic medial septal neurons in hippocampal theta rhythm. Hippocampus.

[CR97] Zhang H, Lin S-C, Nicolelis MAL (2010). Spatiotemporal coupling between hippocampal acetylcholine release and theta oscillations in vivo. J Neurosci.

